# Commentary on “Re-exploration of the optimal threshold for restrictive transfusion after hip fracture: a multicenter prospective cohort”

**DOI:** 10.1097/JS9.0000000000002851

**Published:** 2025-06-25

**Authors:** Xianwen Sun, Jiekun Jian

**Affiliations:** a The Second School of Clinical Medicine, Zhejiang Chinese Medical University, Hangzhou, Zhejiang, China; b The First Affiliated Hospital of Zhejiang Chinese Medical University, Hangzhou City, Zhejiang Province, China

*Dear Editor,*

We have read with great interest the article titled “*Re-Exploration of the Optimal Threshold for Restrictive Transfusion after Hip Fracture: A Multicenter Prospective Cohort*” published in *International Journal of Surgery*^[1]^. We commend the authors for their valuable contribution to optimizing transfusion strategies in hip fracture patients. This multicenter study provides important insights into restrictive transfusion thresholds, particularly for patients with and without cardiovascular disease (CVD). However, after carefully reviewing the study, we believe there are several areas for improvement that warrant further exploration. In line with the TITAN 2025 guideline on AI transparency ^[2]^, a generative AI-based language model was used solely for language polishing and had no bearing on the scientific content.

First, while the study is based on a multicenter prospective cohort, which enhances the applicability of the findings, the lack of randomization leaves room for potential selection bias. A randomized controlled trial (RCT) design would provide more robust evidence. For instance, the FOCUS trial conducted by Carson et al. (2015) demonstrated no significant difference in 60-day mortality between restrictive and liberal transfusion groups in hip fracture patients^[3]^. Future studies should consider adopting an RCT design to reduce bias and verify these findings across various populations.

Second, the study reports that a restrictive transfusion threshold (<7 g/dL) did not increase mortality in patients without CVD. This finding is consistent with the results from Cochrane reviews conducted by Carson et al. (2021), which suggest that restrictive transfusion strategies are safe for patients with hip fractures, reducing both transfusion rates and associated complications^[4]^. However, the authors did not evaluate long-term functional recovery or quality of life, which are crucial outcomes for evaluating transfusion strategies. We suggest that future studies should focus on the long-term effects of restrictive transfusion thresholds, particularly in terms of functional outcomes and patient well-being.

Moreover, the authors observed that for patients with cardiovascular disease (CVD), a restrictive transfusion strategy led to significantly higher mortality (HR 1.45, 95% CI 1.05–1.99, *P* = 0.023). This finding aligns with the work of Mazer et al. (2017), who demonstrated that restrictive transfusion in patients with CVD increases mortality risk due to exacerbated myocardial hypoxia^[5]^. Given these findings, we agree with the authors that maintaining a higher transfusion threshold for patients with CVD seems prudent. However, further research is needed to investigate the underlying mechanisms and explore alternative strategies to mitigate these risks.

The authors also included a subgroup analysis for patients with chronic kidney disease (CKD), which suggested an increased risk of mortality with a lower transfusion threshold (HR 1.90, *P* = 0.079), though this result did not reach statistical significance. This trend is consistent with findings from other studies, such as the work by Sato et al. (2018), who noted that anemia in CKD patients increases mortality risk due to exacerbation of renal ischemia^[6]^. We recommend that future studies expand the sample size and conduct more detailed analyses of this high-risk group to better understand the effects of restrictive transfusion on CKD patients.

One limitation of this study is its relatively short follow-up period, which was limited to 1 year. While the study focuses on mortality, it does not capture the long-term impact of restrictive transfusion on organ function or quality of life. The literature indicates that anemia can lead to organ hypoxia, which may increase the burden on vital organs such as the heart and kidneys, potentially leading to long-term complications. Therefore, we suggest that future studies extend the follow-up period to assess the long-term effects of restrictive transfusion strategies.

Finally, we emphasize the global relevance of this study. With increasing concerns about blood supply shortages, exploring ways to safely reduce transfusion demand is of critical importance. Previous studies have demonstrated the potential for broader adoption of restrictive transfusion strategies in settings with limited blood resources. We encourage further research to explore how these strategies can be implemented in different healthcare settings and assess their feasibility and impact on patient outcomes.

In conclusion, while this study provides valuable data for optimizing transfusion thresholds in hip fracture patients, we believe there are several areas for improvement. Future studies should incorporate randomized controlled trial designs, longer follow-up periods, and more detailed subgroup analyses, particularly in high-risk populations such as those with cardiovascular disease and chronic kidney disease. Moreover, examining the long-term impact of these transfusion strategies on organ function and overall patient well-being will be essential to fully evaluate their clinical utility.

## Data Availability

Not applicable.

## AI assistance

A generative AI–based language model was employed solely for grammar and style editing.
